# CASK, the Soluble Glomerular Permeability Factor, Is Secreted by Macrophages in Patients With Recurrent Focal and Segmental Glomerulo—Sclerosis

**DOI:** 10.3389/fimmu.2020.00875

**Published:** 2020-05-12

**Authors:** Xiaomeng Zhang, Florence Herr, Amelia Vernochet, Hans K. Lorenzo, Séverine Beaudreuil, Antoine Dürrbach

**Affiliations:** ^1^INSERM U1197, Villejuif, France; ^2^Division of Internal Medicine, Department of Nephrology, Tongji Hospital, Tongji Medical College, Huazhong University of Science and Technology, Wuhan, China; ^3^University of Paris-Saclay, Saint-Aubin, France; ^4^Centre de Reference Maladie Rare du Syndrome Nephrotique Idiopatique, Paris, France; ^5^Department of Nephrology, Bicêtre Hospital, Le Kremlin-Bicêtre, France; ^6^Department of Nephrology, Henri Mondor Hospital, Creteil, France

**Keywords:** CASK, focal and segmental glomerulosclerosis, macrophages, exosomes, idiopathic nephrotic syndrome

## Abstract

**Introduction:** Focal and segmental glomerulosclerosis (FSGS) is a frequent form of glomerulonephritis that may be caused by a soluble permeability factor and regulated by the immune system. We previously described a soluble form of calcium/calmodulin-dependent serine/threonine kinase (CASK) acting as a permeability factor in patients with recurrent FSGS (rFSGS). Here, we aimed to identify the immune cells associated with CASK secretion in patients with rFSGS.

**Methods:** FACS, western blotting and immunoprecipitation were performed to detect CASK in peripheral blood mononuclear cells, including CD3^+^, CD20^+^, and CD14^+^subsets, from patients with rFSGS, healthy donors, transplant patients and patients with nephrotic syndrome due to diabetes mellitus, and in KHM2 cells.

**Results:** CASK was produced mostly by monocytes in patients with rFSGS but not by T or B lymphocytes. It was not detectein cells from control patients. CASK was also produced and secreted by M2 polarized macrophages and KMH2 cells, but not by M1 polarized macrophages. CASK secretion was not not inhibited by brefeldin A, suggesting an absence of classical secretion pathway involvement. Within cells, CASK was partly colocalized with ALIX, a molecule involved in exosome development, and these two molecules were coprecipitated from M2 macrophages. Moreover, exosomes derived from M2 macrophages induced podocyte cytoskeleton alterations and increased podocyte motility.

**Conclusion:** These results suggest that the soluble permeability factor CASK is secreted by monocytes and M2 macrophages, via exosomes, to alter the glomerular filtration barrier in rFSGS.

## Introduction

Idiopathic nephrotic syndrome (iNS) is a group of diseases characterized by glomerular lesions and caused by various injuries leading to lesions of the basal membranes and podocytes (1). Focal segmental glomerulosclerosis (FSGS) is a major lesion that can lead to end-stage renal failure. It may occur secondary to hyperfiltration, viral infection, or may be associated with mutations of genes encoding podocyte proteins, such as nephrin, podocin, or alpha-actinin-4 (2). INS could also be associated with ectopic or altered expression of podocyte membrane protein including CD80 or SMPDL-3B that participated directly or indirectly to the regulation of actin cytoskeleton or the recruitment of circulating cells (3, 4). It may also be caused by the secretion of a permeability factor (PF) altering the glomerular basal membrane, characterized by massive proteinuria and hypoalbuminemia. FSGS accounts for 20% of nephrotic syndrome cases in children and 40% in adults and the disease can recur after transplantation in 30–50% of first kidney transplants and up to 90% of second grafts (5–8). Several PFs, including suPAR, cardiotrophin-like cytokine-1, angioprotein-like 4, and anti-CD40 autoantibodies, have been suggested to be implicated in FSGS, but none of these factors has been shown to be related to FGSG recurrence (rFSGS) (9–15). We recently reported that a soluble form of calcium/calmodulin-dependent serine/threonine kinase (CASK) acts as a PF in patients with rFSGS, and that this molecule can be removed by plasma exchange and immunoadsorption on protein A columns (16).

CASK, a member of the membrane-associated guanylate kinase (MAGUK) family, is a scaffolding protein that can link membrane receptors to cytoskeleton proteins, thereby regulating neuronal and epithelial cell polarity (17). CASK is widely expressed in the neuron, kidney and spleen (18). It has been reported to bind to CD98 in the extracellular space of intestinal epithelial cells, thereby modulating amino-acid transport or the organization of the actin cytoskeleton, depending on the coreceptor associated with CD98 (19, 20). In podocytes, the siRNA-mediated silencing of CD98 prevents the cytoskeleton alterations induced by CASK (16).

The cells responsible for releasing this molecule into the plasma of patients with rFSGS have yet to be identified. However, the immune system is thought to play a key role in this disease. iNS can be favored by viral disease or vaccination, but decreases in proteinuria have been observed after measles, which impairs the immune system, in patients with iNS (1, 21, 22). iNS has been also observed in patients with lymphoproliferative diseases (Hodgkin's disease and T-cell lymphoma), disappearing during the remission of these diseases (23, 24). Furthermore, current treatments include drugs targeting both the innate and adaptive immune systems, such as steroids, calcineurin inhibitors and rituximab (3, 25), and c-mip and NF-?? have been shown to be upregulated in T lymphocytes during relapses of the disease (26, 27).

In this study, we aimed to identify the cells producing and secreting CASK in patients with rFSGS, and to decipher the secretion mechanism. By analyzing peripheral blood mononuclear cells from patients with rFSGS, we found that CASK was secreted by monocytes (CD14-positive cells) and M2 macrophages from these patients.

## Materials and Methods

### Patients

#### Group 1

Five patients with rFSGS after renal transplantation were included. The immunosuppressive regimen was thymoglobulin for induction, and tacrolimus, mycophenolate mofetil, and corticosteroids for maintenance therapy. Proteinuria recurred in all patients, and graft biopsy was performed to rule out acute rejection (Table 1). Renal biopsies showed minimal change disease or FSGS. The patients were treated with high-dose steroids and tacrolimus (with a tacrolimus trough level >13 ng/mL) for the recurrence of FSGS. Peripheral blood was collected from all patients at the time of recurrence.

**Table 1:** Patient's description.

	**rFSGS**	**Stable Transplant Patient**	**Nephrotic syndrome**	**Healthy donors**
Number	5	7	5	8
Age (year)	39.8 ± 7.3	53.7 ± 16.5	66.7 ± 6.4	44.2 ± 9.1
Sexe F/M	1/4	2/5	1/4	3/5
Kidney disease	Recurrent FSGS	• IgA-GN (*n* = 1) • Alport syndrome (*n* = 2) • NAS (*n* = 2) • Diabetes Type 2 (*n* = 1) • Unknown (*n* = 1)	Diabetes type 2	na
Hypertension (number)	5	7	5	0
Serum creatinine (μmol/l)	150.5 ± 101	202 ± 98	288 ± 162	na
Proteinuria (g/day)	6.8 ± 2.4	0.3 ± 0.22	4.9 ± 1.9	na
Time between Transplantation and Proteinuria (days)	22.8 ± 21.6	na	na	na
Time between Transplantation and blood samples (days)	27.8 ± 25	61.2 ± 32.1	na	na
Dialysis	No	No	No	na
Kidney transplant	5	7	No	na
CNI treatment	5	5	No	na

*Na, non-applicable, NAS, nephroangiosclerosis, IGA-GN, IgA associated glomerulonephritis. CNI, calcineurin inhibitor*.

#### Group 2

Seven kidney-transplant patients without FSGS were included. The causes of nephropathy in these patients were diabetes mellitus (*n* = 2), IgA nephropathy (*n* = 3), or unknown (*n* = 2). All had end-stage renal disease and had undergone transplantation. The patients were treated with tacrolimus, mycophenolate mofetil, and steroids. Their renal function was stable, with no acute or chronic rejection.

#### Group 3

Five patients with chronic kidney failure and nephrotic syndrome caused by type 2 diabetes were included. All had biopsy-proven diabetes associated with glomerulonephritis. Peripheral blood was collected from all patients.

#### Group 4

Peripheral blood samples were collected from eight healthy donors.

The project was approved by the local ethics committee ≪ Comité Consultatif de Protection des Personnes participant à une Recherche Biomédicale ≫ (n°4/010). All patients provided their written informed consent (patients from groups 1, 2, and 3). Healthy donors samples were collected by Etablissement Français du Sang after written informed consent. The informatic file developed for the research was approved by the national commission of informatic and liberty. None of the transplant donors were from a vulnerable population and none of them had declared their opposition for organ procurement accordingly to French law (Loi de Bioethique Article L. 1232- 1).

### Reagents and Antibodies

#### Production of Recombinant CASK

The cDNA sequence for human CASK was kindly provided by Prof. Zenta Walther (Yale University, School of Medicine). The DNA was digested with *Bam*HI and *Eco*RI and inserted into the pTrcHis2C vector (Invitrogen) for expression and purification of the protein product in *Escherichia coli*. Gene expression and protein purification were performed by Genscript Services (Piscataway, NJ, USA). Briefly, the recombinant protein was purified by affinity chromatography on Ni-agarose columns followed by anion-exchange chromatography. The final purity was close to homogeneity (>95%) and endotoxin levels were below 0.10 EU.

Human IL-4 and M-CSF were obtained from ImmunoTools (Friesoythe, Germany) and human recombinant IFNγ was purchased from Miltenyi Biotec (BergischGladbach, Germany). Lipopolysaccharide and brefeldin A were purchased from Sigma-Aldrich (Saint-Louis, USA). Short interfering RNA (siRNA) sequences directed against human CASK were purchased from OriGene (Rockville, USA). The antibodies against CASK used for western blotting were from Santa Cruz Biotechnology (Dallas, USA) (H107, targeting amino acids 353–459) or from BD Biosciences (610782, targeting amino acids 353–486). The anti-CASK antibody used for Flow cytometry and microscopy was from Abcam (Cambridge, UK, ab126609). Antibodies against ALIX and LAMP2, were purchased from Abcam. Antibodies against actin and synaptopodin were purchased from Santa Cruz Biotechnology. Antibody against GAPDH was purchased from Sigma-Aldrich. Antibodies against Calnexin, GM-130, CD3-APC and CD3-FITC, CD20-APC, CD14-APC, and CD14-PE-Cy7, CD206-PE-Cy7, CD163-PE and isotype controls were purchased from BD Biosciences. Horseradish peroxidase-conjugatedsecondary antibodies for western blotting, or fluorescent conjugated secondary antibodies for immunofluorescence analysis were purchased from Jackson ImmunoResearch (West Grove, USA). Antibody against CD63 was kindly provided by Dr. Eric Rubinstein (INSERM).

### Cell Culture

#### Podocyte

A conditionally immortalized mouse podocyte cell line was cultured, as described by Mundel et al. (28). Briefly, non-differentiated podocytes were cultured in RPMI supplemented with 10% fetal calf serum (FSC), 2 mM L-glutamine, 100 U/ml penicillin/streptomycin (Invitrogen, California,USA), 50 U/ml IFNγ (for the first two passages, and 10 U/ml IFNγ thereafter) at 33°C under an atmosphere containing 5% CO_2_. Differentiation was induced by treating the podocytes with trypsin and culturing them in the same medium, without IFNγ, for 2 weeks at 37°C.

In experiments with cells microvesicules isolation, microvesicles were removed from the FCS by centrifugation at 100 000 × g for 2 h before addition to the culture medium.

#### Macrophage Polarization

Peripheral blood mononuclear cells (PBMCs) were isolated from healthy donors by Ficoll-Paque density-gradient centrifugation (GE Healthcare Life Sciences, Buckinghamshire, UK). Monocytes were isolated from PBMCs by two passages of adherence on plastic culture plates. Purity of monocyte were assessed by flow cytometry with anti-CD14mAbs (ImmunoTools, Friesoythe, Germany). Monocytes were maintained in culture at a density of 5 × 10^5^/cm^2^, in RPMI 1640 supplemented with 10% FCS, 2 mM L-glutamine, and 100 U/ml penicillin/streptomycin. M1-polarized macrophages were obtained by culturing the monocytes for 7 days with 1000 U/ml IFNγ. M2-polarized macrophages were obtained by culturing the monocytes for 7 days with 50 ng/ml M-CSF and 10 ng/ml IL-4. Cells displaying intermediate differentiation were cultured in the same medium supplemented with 50 ng/ml M-CSF for 7 days at 37°C, under an atmosphere containing 5% CO_2_.

#### PBMCs Isolation

PBMCs were isolated from the blood of healthy volunteers, rFSGS patients, kidney-transplant patients and type 2 diabetes patients by density Ficoll-Paque gradient centrifugation. Briefly, blood collected in EDTA-coated tube was diluted in Hanks Balance Salt Solution (HBSS) at 1:1 ratio and layered on Ficoll-Paque plus solution (GE Healthcare Life Sciences, Buckinghamshire, UK) (1 volume Ficoll-Paque: 2 suspension volumes). After centrifugation 400 g 30 min at room temperature the layer of mononuclear cells was transferred in a tube and washed two times in 10 volumes HBSS.

### Exosome Purification

Exosomes were purified from cell supernatants by three successive centrifugations: an initial centrifugation at 10 000 × g (30 min) to eliminate cells and debris, followed by a concentration step with amicon Ultra centrifugal filter (Merk) at 4 000g and then an ultracentrifugation for 2 h at 100 000 × g. The exosome pellet was washed once in a 5 mL of PBS, centrifuged at 100 000 × g for 2 h and the resulting pellet was then resuspended in 50–200 μl PBS.

We used a modified version of the classical protocol to obtain plasma microvesicles, due to the viscosity of the plasma and the higher abundance of lipids in the plasma than in the cell supernatant. Plasma was centrifuged for 30 min at 500 × g, 45 min at 12 000 × g and 2 h at 100 000 × g. Pellets were resuspended in 5 mL of PBS, and the suspension was passed through a filter with 0.22 μm pores(Millipore, Massachusetts, USA) and centrifuged at 100 000 × g for 2 h. The resulting microvesicle pellets were washed once in 5 mL of PBS, centrifuged at 100 000 × g for 2 h and the final pellet was resuspended in 50–200 μl PBS.

Protein concentrations were determined with the BCA protein assay kit (Thermo Fisher Scientific, Massachusetts, USA) for immunocytochemistry, immunoblotting or functional assays. Exosomes were used as fresh preparations or after freezing and storage at −80°C.

### Flow Cytometry

#### Cells

PBMCs isolated from healthy donors, patients with rFSGS, transplant patients without FSGS or from patients with nephrotic syndrome were analyzed. PBMCs were isolated from whole blood by Ficoll-Paque (GE Healthcare Life Sciences) density-gradient centrifugation. Phenotypic analyses were performed on the various subpopulations of PBMC as follows: cells were incubated with labeled primary antibody directed against a membrane protein or isotype control, and were then washed in PBS and fixed by incubation in 3% paraformaldehyde (PFA) for 30 min. After washing in PBS, Fc receptors were saturated by incubation with 10% AB-human serum (Life Technologies, California, USA). For the subsequent intracellular staining of CASK, cells were washed in PBS, incubated for 15 min in 100 mM NH_4_Cl in PBS, permeabilized by incubation with 0.1% saponin in 0.2% BSA and stained with specific primary antibodies followed by secondary antibodies (goat anti-rabbit AlexaFluor-488). Anti-CD3, anti-CD20, and anti-CD14mAbs were purchased from ImmunoTools and rabbit anti-CASK antibodies were obtained from Abcam (Cambridge, USA). Cells were analyzed with aFACScalibur™ machine (BD Biosciences, Franklin, USA), with Cell Quest Analysis (BD Biosciences) and FlowJo (BD Lifesciences) software.

#### Exosomes

For FACS analysis, exosomes were incubated with sulfate latex beads (Life Technologies) at a ratio of 2 μg exosomes to 10 μl beads. The volume was made up to 500 μl with PBS and the mixture was incubated for 2 h at room temperature. The beads were blocked by incubation with glycine (100 mM) for 30 min and were then washed three times with 0.5% BSA in PBS. The exosome-coated beads were resuspended in 500 μl PBS. We then incubated 10 μl of bead suspension with the primary antibodies (anti-CASK, anti-CD9, anti-CD63, and anti CD81) at a dilution of 1/50, followed by the secondary antibody (goat anti-rabbit PE, goat anti-mouse APC; Jackson Immuno Research, Cambridgeshire, UK). FACS analysis was performed on a BD Accuri C6 flow cytometer with CFlow plus software (BD Lifesciences).

### Immunofluorescence

#### Immunofluorescence of Cells

Podocytes were grown on coverslips and incubated with recombinant CASK or macrophage-derived exosomes for 24 h. For immunofluorescence staining, cells were washed three times in PBS and fixed by incubation with 3%PFA in PBS for 20 min. They were then washed in PBS and incubated for with 100 mM NH_4_Cl in PBS for 10 min. Cells were permeabilized with 0.01% saponin, and then blocked by incubation with 3% BSA-0.01% saponin buffer for 1 h. Finally, the cells were incubated with the primary antibodies for 1 h at room temperature and washed three times before incubation with secondary antibodies or phalloidin 488. Cells were mounted in Mowiol 4–88 medium, and fluorescence was observed with a Leica DM-RXA23D microscope or Leica DM confocal microscope (Wechsler, Germany).

### Gene silencing

For the CASK knockdown experiments, KM-H2 cells were transiently transfected, by electroporation, with a pool of three target-specific human siRNA (100 pmol) oligonucleotides against CASK according to the manufacturer's protocols (Lonza Amaxa, Basel, Germany).

### Videomicroscopy

Cell motility analysis was performed by time-lapse video microscopy on an inverted microscope equipped with a 37°C chamber, under an atmosphere containing5% CO_2_ (Nikon, Tokyo, Japan). Stacks of phase-contrast images were collected every 15 min for 24 h, at ×200 magnification. Cell migration was quantified with the manual tracking plug-in of ImageJ. Data were transferred to Excel for calculations and statistics. For each position, we analyzed at least 10 cells.

### Immunoprecipitation

The cell culture supernatant was concentrated with Amicon ultracentrifugation filter units (Millipore, Massachusetts, USA) and precleaned by incubation with protein G-Sepharose (GE Healthcare Life Sciences, Buckinghamshire, UK) beads for 2 h at 4°C. The supernatant was incubated overnight with anti-CASK antibody (Santa Cruz, H-107) in a rotating mixer at 4°C. Protein G-Sepharose beads were then added and the mixture was incubated for 2 h at 4°C. It was then centrifuged and the beads were washed. The complexes were recovered in 2× Laemmli buffer (BioRad, California, USA) and analyzed by SDS-PAGE.

For co-immunoprecipitation, cells (1.5 × 10^7^) were lysed by incubation in ice-cold lysis buffer (25 mmol/L HEPES, 150 mmol/L NaCl) supplemented with 1% Brij 97 and a protease inhibitor cocktail (Thermo Fisher Scientific, Massachusetts, USA) for 1 h at 4°C. The lysate was centrifuged (10 min, 20000 × g), and 1 mL of the supernatant was then precleared by incubation with Protein G-Sepharose for 2 h at 4°C. The precleared lysates were incubated overnight at 4°C with 1 μg antibody. They were then incubated with Protein G-Sepharose for 2 h, and the immune complexes were washed five times in the lysis buffer. Immunoprecipitated proteins were analyzed by SDS-PAGE and western blotting.

### Immunoblotting

Equal amounts of protein (5–30 μg) or immunoprecipitate were subjected to SDS–PAGE under reducing conditions. The resulting bands were electrophoretically transferred onto a PVDF membrane. They were then fixed in 3% acetic acid, and the membrane was saturated by incubation with 5% BSA in TBS-Tween (0.1%) and incubated with primary mouse antibodies against CASK, β-Actin, GAPDH or ALIX for 1 h at room temperature. The membrane was washed three times in TBS-Tween and incubated with horseradish peroxidase-conjugated secondary antibodies for detection by enhanced chemiluminescence (WBKLS0500, Substrate HRP Immobilon, Millipore SAS, Saint Quentin en Yvelines, France). Protein content was quantified with ImageJ software. The density of the bands was normalized against a reference protein (β-Actin or GAPDH).

## Statistics

Quantitative values were compared in non-parametric Mann-Whitney U tests or ANOVA, qualitative values were compared in Chi^2^ tests. We considered *p*-values below 0.05 to be significant.

## Results

### Expression of CASK by PBMCs in rFSGS Patients

**Figure 1:**
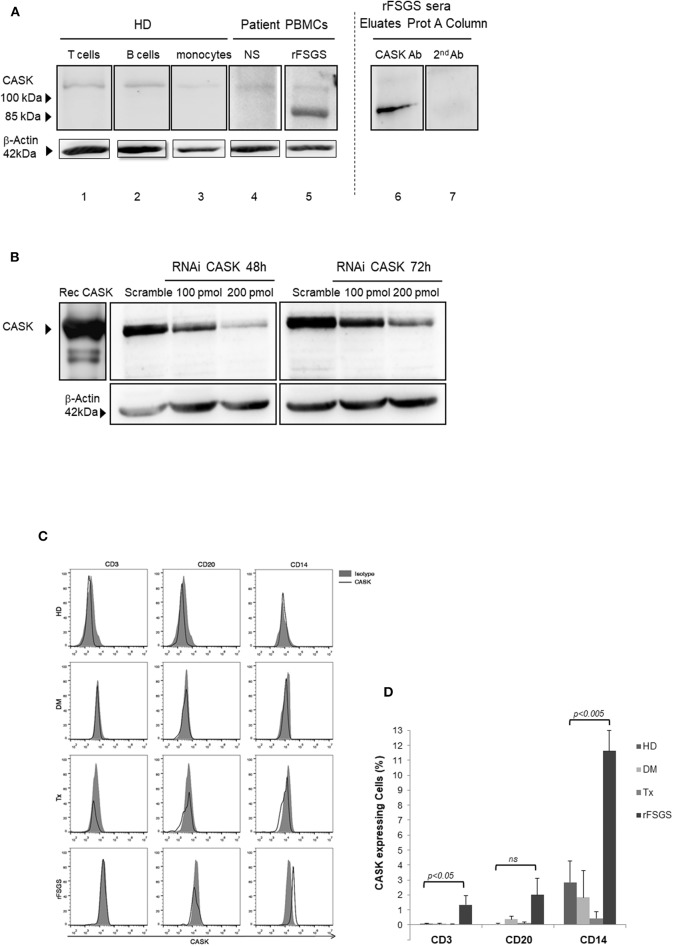
CASK expression in PBMCs. **(A)** (lanes 1–3) T cells, B cells, and monocytes isolated from healthy donors (HD), (lanes 4,5) peripheral blood mononuclear cells (PBMCs) from patients with nephrotic syndrome (NS) due to diabetes mellitus or rFSGS patients, (lane 6,7) eluate from a patient treated with a protein A column, analyzed by immunoblotting with a commercial rabbit polyclonal anti-CASK antibody (lanes 1–6) or a secondary anti-rabbit IgG antibody (lane7). **(B)** Gel electrophoresis and western blotting with an anti-CASK antibody of recombinant CASK (rec CASK) or lysates of KM-H2 cells transfected with an anti-CASK or scrambled siRNA, at various time points. **(C)** Flow cytometry analysis of PBMCs from healthy donors (HD), patients with NS due to diabetes mellitus-associated nephropathy kidney transplant recipients without rFSGS (Tx), or rFSGS patients (rFSGS). Cells were labeled with anti-CASK antibody and either an antibody against CD3, CD20, or CD14. **(D)** Percentages of CASK^+^ cells in the CD3^+^, CD20^+^, and CD14^+^subsets were compared with those in healthy individuals (*n* = 8), diabetic patients (*n* = 5), kidney transplant recipients (*n* = 7) and rFSGS patients(*n* = 4). Statistical differences were determined by ANOVA test for CD3, CD20, and CD14 cells populations (dotted line), and by unpaired student's *t*-test for patient groups among cells populations (solid line).

We investigated CASK levels in PBMCs by western blotting, to determine whether these cells might be responsible for CASK secretion. The immunoblot detected low levels of CASK, with an apparent molecular mass of 105 kDa in T cells, B cells and monocytes from the healthy donor (Figure 1A). In PBMCs from rFSGS patients, we detected CASK with a different apparent molecular mass, ~90 kDa (Figure 1A), consistent with the size of CASK in the serum of patients with rFSGS (Figure 1A). KM-H2 cells, which are derived from a Hodgkin lymphoma, expressed constitutively CASK. We demonstrated the specificity of the CASK antibody by knocking down CASK expression in KM-H2 cells (Figure 1B).

For identification of the subpopulation of cells expressing CASK in patients, we performed FACS with antibodies specific for surface markers of leukocytes (CD3, CD20, and CD14) and intracellular staining for CASK. Significant CASK expression relative to healthy patients was detected in 11.6 ± 1.4% of CD14^+^ cells from patients with rFSGS (*p* < 0.0001). Expression of CASK was also detectable in a small fraction of T cells (1.3 ± 0.6%) and B-lymphocytes (2.8 ± 1.1%) of rFSGS patients as compared to the other groups of patients (Figures 1C,D). CASK was not significantly detected in PBMCs from transplant patients or from patients with nephrotic syndrome due to diabetes mellitus glomerulonephritis. Comparing expression of CASK between CD14+ cells of rFSGS patients and those from patients with diabetes mellitus or transplant patients or healthy donors, we observed a higher expression in rFSGS CD14+ cells (Figure 1D). In addition, the fraction of cells expressing CASK in PBMC of rFSGS patients were substantially higher in the CD14 population than in T or B lymphocytes populations. Thus, we analyzed CASK expression by CD14+ derived cells.

### CASK Expression in the M2 Macrophage Subset

**Figure 2:**
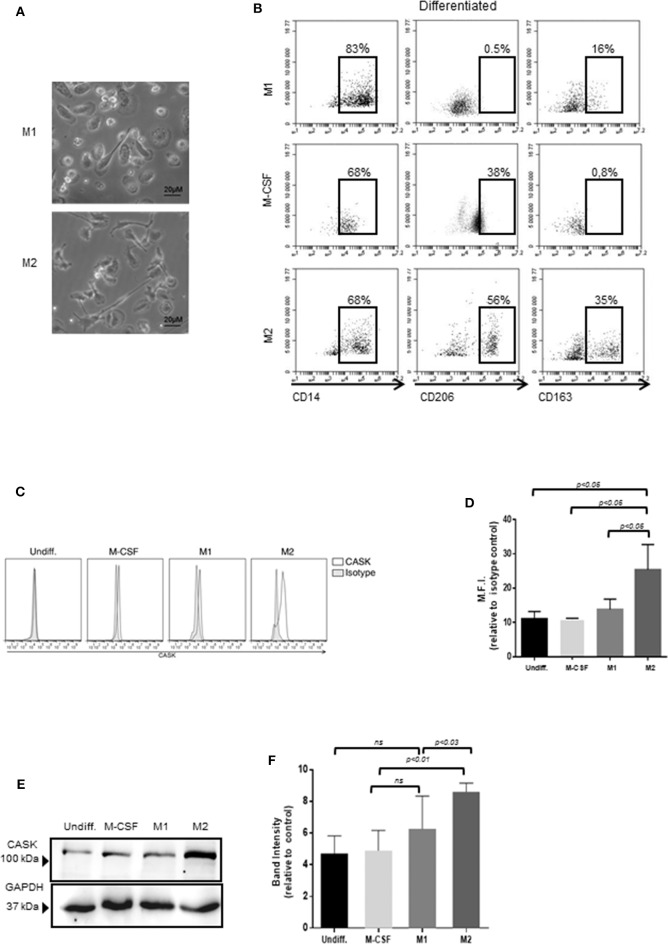
Expression of CASK in the macrophage subsets. **(A)** The morphology of M1 and M2 macrophages was analyzed by phase-contrast microscopy with a ×20 objective. **(B)** Surface marker expression of M2, M1 and undifferentiated macrophages (CD14, CD206, CD163) was analyzed by flow cytometry (*n* = 4). **(C,D)** CASK expression of macrophage subsets was evaluated by intracellular staining and flow cytometry analysis and mean fluorescence intensity (M.F.I) was quantified, relative to isotype control (*n* = 4) **(E)** CASK expression was assessed by SDS-PAGE and immunoblot analysis in the different macrophage subsets. **(F)** Quantification of the intensity of the CASK band with Image J software (*n* = 4).

Monocytes/macrophages constitute a heterogeneous population that can be separated into different subsets on the basis of cell development and phenotype. We investigated the macrophage subsets involved in CASK production, by promoting the differentiation of monocytes purified from healthy individuals and their polarization into M1 or M2 macrophage subpopulations (Figure 2A). The cells of the M2 subset were elongated, with a fibroblast-like morphology, contrasting with the classical “fried egg shape” of the cells of the M1 subset (Figure 2A). Furthermore, the cells of the M2 subset strongly expressed the prototypic markers CD206 and CD163, which were absent from the M1 subset (Figure 2B). The monocytes treated with M-CSF had an intermediate phenotype. FACS demonstrated stronger CASK expression in M2 macrophages than in M1 macrophages (Figures 2C,D). These results were confirmed by western blotting (Figures 2E,F).

### Secretion of CASK by M2 Macrophages

**Figure 3:**
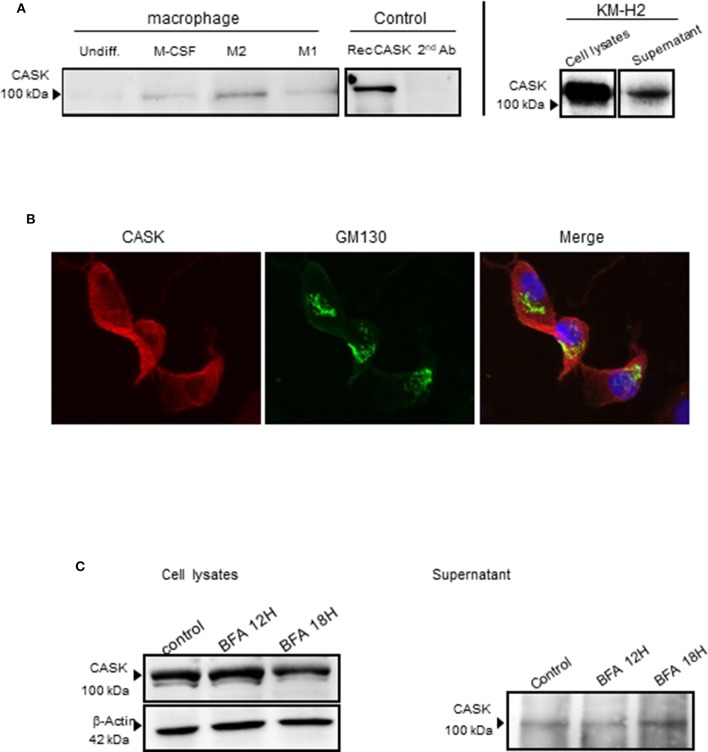
CASK secretion by the macrophage subsets and KM-H2s. **(A)** Immunoprecipitation of CASK, with a rabbit polyclonal Ab, from the supernatant of different macrophage cultures and recombinant CASK, followed by immunoblotting with mouse anti-CASK antibody **(B)** M2 macrophages were stained with antibodies against CASK (red) and GM130 (green), a *cis*-Golgi matrix protein, ×63 magnification. **(C)** Protein transport from the endoplasmic reticulum to the Golgi apparatus was blocked by treating KM-H2 cells with brefeldin A (10 μg/ml) for 12 or 18 h. The production and secretion of CASK were evaluated by western blots of cell lysates and supernatants, respectively.

We then investigated the secretion of CASK by these cells. We subjected the supernatants of KM-H2 cells and macrophages to immunoprecipitation for CASK before subjecting gel electrophoresis and western blotting. CASK was detected in the supernatant of KM-H2 cells, and in the supernatant of M2 cells, but much less strongly in the supernatant of M1 cells (Figure 3A).

### Subcellular Localization of CASK

CASK is a membrane-associated cytoplasmic protein with no signal peptide. We therefore investigated the mechanism of CASK secretion in KM-H2 cells, in which CASK is cytoplasmic and not colocalized with GM 130, a *cis*-Golgi protein (Figure 3B). We investigated the role of the classical pathway in CASK secretion, by treating KM-H2 cells with brefeldin A (BFA), a fungal metabolite that inhibits ER-to-Golgi trafficking. BFA did not inhibit the secretion of CASK or induce its intracellular accumulation (Figure 3C), suggesting the involvement of another release pathway.

**Figure 4:**
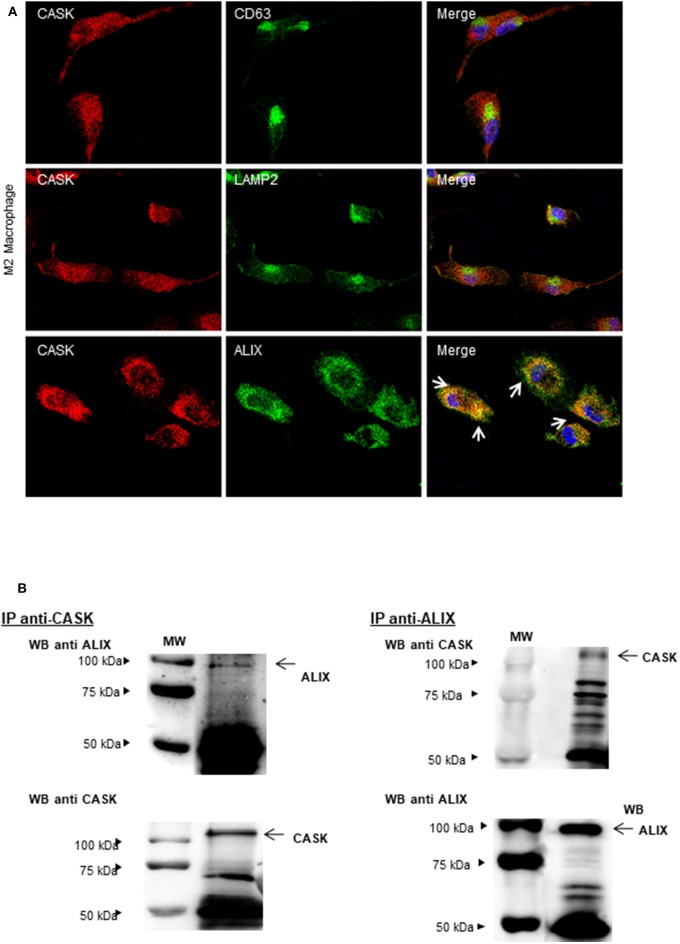
Subcellular distribution of CASK and interaction between CASK and ALIX. **(A)** M2 macrophages were stained with antibodies against CASK and CD63, CASK, and LAMP2 or CASK and ALIX, and then with secondary antibodies labeled with Alexa Fluor 488 and Alexa Fluor 594. The samples were analyzed by confocal microscopy, with a ×63 objective. The co-distribution of two markers is indicated by arrows in the composite image. **(B)** Reciprocal co-immunoprecipitation was performed with antibodies against CASK and ALIX, in M2 macrophages. The immunoprecipitates were analyzed by western blotting with mAbs against CASK or ALIX (*n* = 3). IP: immunoprecipitation.

Exosomes provide an alternative pathway for protein secretion. They are of endosomal origin and are formed by the inward budding of multivesicular bodies (MVBs) (29). We therefore investigated the distribution of CASK in these compartments by confocal microscopy with antibodies against CASK, CD63 (a late endosomal marker), LAMP2 (a lysosomal marker) or ALIX (an auxiliary component of the ESCRT: endosomal sorting complexes required for transport) (30) in the M2 macrophage subset. CASK had a diffuse cytosolic distribution (Figure 4A). It was not colocalized with CD63 or LAMP2. Partial colocalization was observed between CASK and ALIX. We then performed co-immunoprecipitation experiments with Brij97 detergent, to investigate the association of CASK with ALIX further. ALIX was co-immunoprecipitated with CASK and, conversely, CASK was co-immunoprecipitated with ALIX (Figure 4B). These results confirmed association between these two proteins.

### Exosome-Associated CASK Release

**Figure 5:**
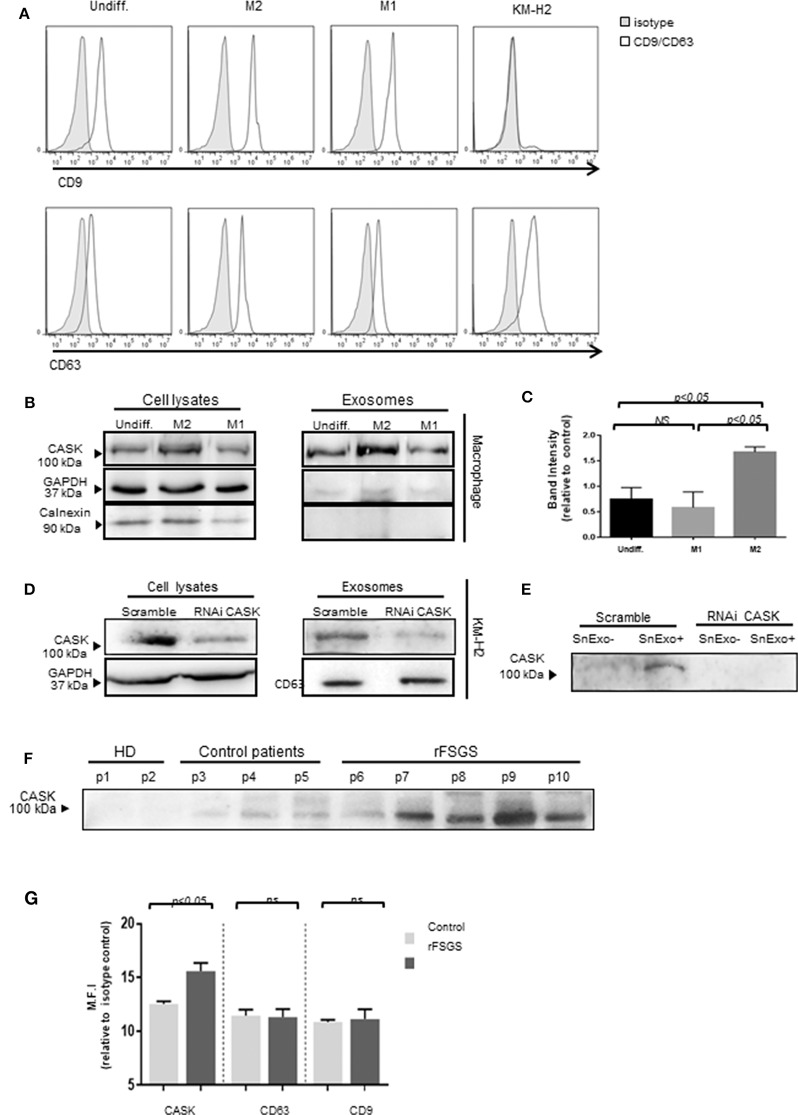
Exosome-associated CASK release. Exosomes were purified from culture supernatants of KM-H2 cells and M1 and M2 macrophages, by differential ultracentrifugation. **(A)** Surface exosomal marker expression was analyzed by flow cytometry for CD9 and CD63 expression. **(B,D)** CASK levels were investigated in cell lysate or exosomes from macrophage subsets **(B)** and from KM-H2 cells transfected with a siRNA directed against CASK or with a scrambled sequence as a negative control **(D)** (*n* = 3). Total cell lysates and corresponding exosomes were analyzed by immunoblottingwith anti-CASK, anti-GAPDH or anti-calnexin antibodies. **(C)** CASK band intensity of **(B)** was quantified with Image J software, with normalization against GAPDH (*n* = 4). **(E)** Immunoblotting with anti-CASK antibody of exosome-containing supernatant (SnExo^+^) and exosome-depleted supernatant (SnExo^−^) from KM-H2 cells of **(D)** (*n* = 4). **(F)** Exosomes were purified from the plasma of healthy controls (HD) (P1–P2), control patients with NS due to diabetes mellitus (P3–P5) or rFSGS patients (P6–P10). CASK levels in these exosomes were analyzed by western blotting. **(G)** The expression of CASK, CD63, and CD9 in control patients (*n* = 4) and in rFSGS patients (*n* = 5) was analyzed by flow cytometry. Mean fluorescence intensity for CASK, CD63 andCD9 relative to isotype control, displayed as a histogram.

To investigate the association of CASK with exosomes, we purified exosomes from the supernatant of M1 or M2 macrophages and subjected them to FACS analysis. We detected high levels of expression for exosomal markers, such as CD9 and CD63 (Figure 5A) in exosomes from macrophages and only CD63 in those purified from KM-H2 cells. None of the exosome preparations tested displayed an association with calnexin (Figures 5B,C), an ER membrane component. In KM-H2 cells, CASK was detected in the exosomal fraction (Figure 5D), mostly in exosomes from M2 subset. Quantification of the CASK/GAPDH density ratio revealed significantly higher levels of CASK expression in exosomes from the M2 subset (Figure 5C) than in those from the M1 fraction or undifferentiated macrophages. In KM-H2 cells, CASK knockdown with siRNA efficiently reduced CASK levels in both the cell lysate and exosomes (Figure 5D). For confirmation that the secreted CASK was mostly associated with exosomes, we compared CASK levels in the supernatant of KM-H2 cells or macrophage subsets before and after exosome removal. As expected, CASK was detected in the supernatant (SnExo^+^) of KM-H2 cells, but not in the supernatant of these cells after ultracentrifugation to remove the exosomes (SnExo-) (Figure 5E). CASK was also not detected after its knock down with a specific siRNA. These results suggest that CASK release may be associated with exosomes in the cellular microenvironment.

### Expression of CASK in the Plasma-Derived Exosomes of rFSGS Patient

We then investigated whether CASK was presenting exosomes from rFSGS patients. For this purpose, we purified exosomes from the sera of rFSGS patients, healthy controls and transplant patients without proteinuria. CASK was present in exosomes from rFSGS patients, but not in those from healthy donors, as shown by immunoblotting (Figure 5F). It was detected in control patients, but to a lesser degree. These results were confirmed by flow cytometry. For this purpose, exosomes were fixed on microbeads (see methods). Flow cytometry analysis revealed significantly higher levels of CASK expression on the exosomes from patients with rFSGS than on those from control patients (*p* < 0.05), whereas CD63 and CD9 levels were similar for the two groups (Figure 5G).

### M2-derived Exosome-Induced Podocyte Alterations

#### Cytoskeleton Alterations

**Figure 6:**
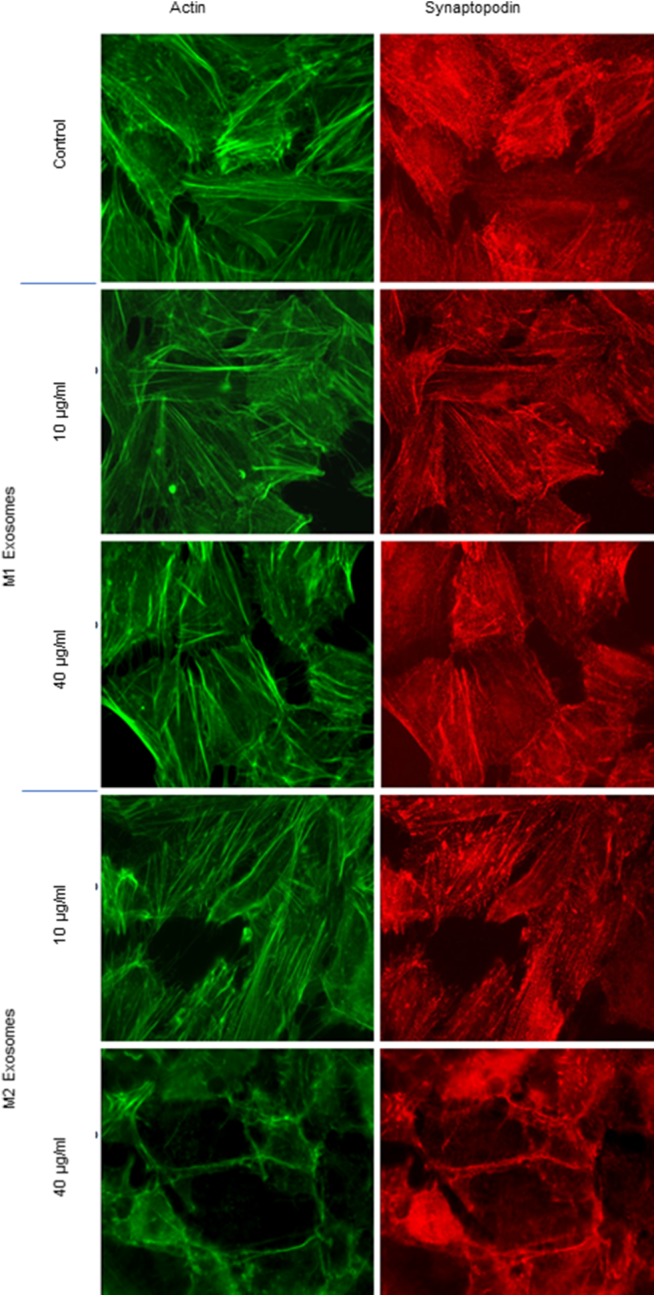
Cytoskeleton alterations induced by exosomes from M1 or M2 macrophages. Exosomes from M1 and M2 macrophages were incubated with human podocytes at concentrations of 10 and 40 μg/ml. Immunostaining was performed with phalloidin (green) and anti-synaptopodin antibody (red). Analysis was performed by fluorescence microscopy with a ×63 objective.

CASK induced cytoskeleton alterations in podocytes (16). We therefore investigated the impact of exosomes purified from macrophage subsets on the morphology of human podocytes. M2-derived exosomes induced a dose-dependent loss of actin stress fibers, with maintenance of the peripheral organization of F-actin (Figure 6). Similarly, exosomes affected synaptopodin, an actin-associated protein displaying liner codistribution with actin filaments in control cells (Figure 6). In the presence of M2-derived exosomes, synaptopodin staining was diffuse. By contrast, treatment with 40 μg/ml M1-derived exosomes had no effect on actin cytoskeleton organization (Figure 6).

#### Increase in Podocyte Motility

**Figure 7:**
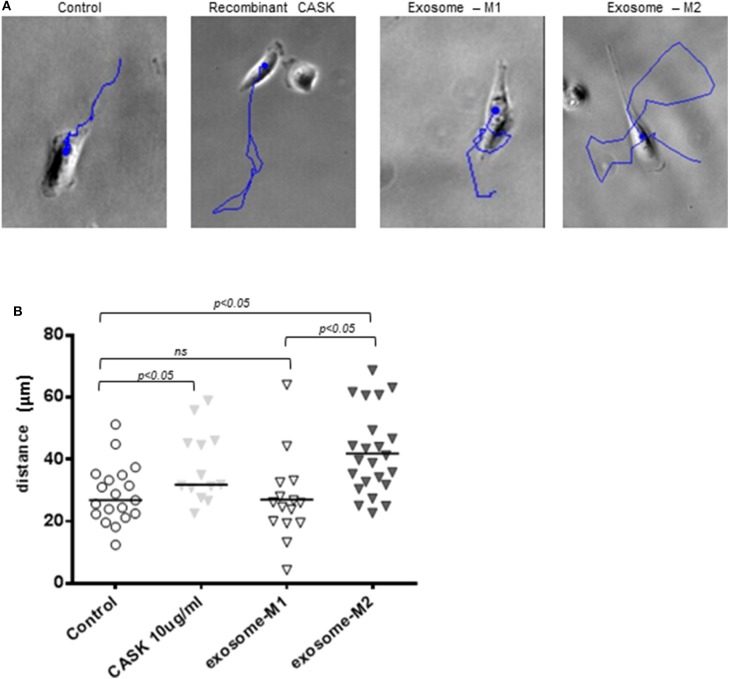
Increase in podocyte motility induced by exosomes from M2 macrophages. Podocyte motility was evaluated by video microscopy for 24 h in the presence of recombinant CASK, M1 exosomes or M2 exosomes. **(A)** Blue lines show the displacement of single cells. **(B)** Image J analysis showing the distance migrated by podocytes. Statistical differences were determined by ANOVA test.

We investigated whether the disruption of the actin cytoskeleton observed after the addition of exosomes from the M2 subset was associated with a functional effect on podocytes, through video microscopy explorations of podocyte motility. Podocytes were grown on plates coated with collagen IV and treated with recombinant CASK, M1-derived exosomes or M2-derived exosomes. Both CASK and M2-derived exosomes induced an increase in podocyte motility (5.5 ± 1.531 μm/h and 6.8 ± 1.265 μm/h, respectively) relative to control podocytes (4.66 ± 1.068 μm/h) or podocytes treated with M1-derived exosomes (4.16 ± 1.075 μm/h)(Figures 7A,B; *p* < 0.05).

## Discussion

In a previous mass spectrometry study analyzing the proteins eluted from protein A columns used to treat rFSGS, we identified a serum form of CASK in rFSGS patients. Recombinant CASK induced the impairment of podocytes *in vitro* (cytoskeleton alterations) and proteinuria and podocyte foot-process effacement in mice (16). Moreover, as our work suggests rFSGS may be a systemic disease involving the immune system. In this study, we found that CASK was expressed by CD14^+^ cells from patients with rFSGS, but not by those of healthy donors or control patients treated with similar immunosuppressive drugs.

Given the frequency of its recurrence after transplantation, rFSGS is considered to correspond to a systemic disease with a specific effect on glomeruli. Its sensitivity to immunosuppressors and its recurrence after vaccination or viral infection have led to the hypothesis of a role for the immune system in initiating or maintaining the disease. The work of Grimbert et al. supported this view, by demonstrating an upregulation of c-mip in T cells and podocytes from patients with rFSGS (26). In addition, several molecules have been demonstrated to be ectopically expressed or up regulated suggesting that it could be direct or indirect target of the immune system (3, 4). Although, the expression of CD80 is controversial during iSN, this molecule could participate to the interaction of podocytes and the immune system during iSN (31, 32). However, biopsies performed during active processes of rFSGS did not find infiltration of inflammatory cells but we cannot formally ruled out that transient interaction can take place directly or through cell mediators as microvesicles. For these reasons, we investigated the ability of immune system cells to produce CASK in patients with rFSGS, by analyzing CASK production by PBMCs. We found that a fraction of monocytes expressed CASK. Monocytes have a short half-life in blood, but serve as the precursors of macrophages and dendritic cells, which play key roles in innate and adaptive immunity (33, 34). *In vitro*, CASK was produced by M2 macrophages expressing CD206 and CD163. This finding is consistent with the expression of CASK in the spleen and with the demonstration ofTh2 polarization and activation during the development of iNS in Buffalo/Mna rats with minimal change lesion of glomeruli (35, 36).

Soluble CASK was detected in the supernatants of M2 macrophages and KM-H2 cells. It was not associated with markers of cell culture necrosis (data not shown), suggesting that it was secreted by PBMCs. However, CASK has no signal peptide, which suggests that it may not be secreted by the classical secretory pathway. This was indeed confirmed by the absence of CASK co-distribution with secretory pathway organelles, such as the Golgi apparatus and endoplasmic reticulum. Moreover, CASK secretion in KH-M2 cells was not affected by brefeldin A treatment, which blocks endoplasmic reticulum-to-Golgi apparatus transport.

We hypothesized that CASK might be secreted in exosomes, leading to its release into the extracellular environment. Exosome release is an alternative protein secretion pathway that has been suggested to play a major role in the release of IL-1β from murine bone marrow-derived macrophages (37). We detected CASK in exosomes purified from KM-H2 cells and different macrophage subsets. CASK levels were much lower (barely detectable) in the exosome-depleted supernatant than in the supernatant containing exosomes. This suggests that CASK secretion may be associated with exosomes. Before their release into the extracellular medium, exosomes accumulate in MVBs formed by the inward budding and scission of vesicles from the limiting membrane of late endosomes into the lumen. During this process, transmembrane and peripheral membrane proteins are incorporated into the invaginated membrane, whereas cytosolic components are engulfed and enclosed in the vesicles. CASK, which is known to be a membrane-associated cytoplasmic scaffold protein, had a diffuse cytoplasmic distribution pattern not restricted to endosomal compartments. Co-immunoprecipitation methods showed that a fraction of CASK was associated with ALIX. ALIX is connected to syndecans, providing a support for the packaging of cargoes for vesicle entry and triggering vesicle formation (38). CASK has been reported to bind to syndecan (17, 39). Thus, CASK may be recruited to exosomes through binding to ALIX and syndecan.

CASK was also detected in exosomes purified from patients with iNS, and was present in significantly larger amounts in exosomes from the rFSGS group and from M2 macrophages. This higher level of CASK in exosomes was correlated with higher levels of CASK in the cytoplasm of M2 macrophages *in vitro*, suggesting that the higher levels of CASK secretion are mostly due to higher levels of CASK production in the corresponding cells rather than an increase in the rate of transfer of CASK to exosomes.

We previously showed that recombinant CASK induced a reorganization of the actin cytoskeleton of cultured podocytes. M2 macrophage-derived exosomes caused a loss of actin stress fibers and the redistribution of synaptopodinin the podocytes *in vitro*. The depletion of exosomes from the supernatant of M2 macrophages or KM-H2 cells (data not shown) was associated with an absence of change in actin stress fiber organization and synaptopodin redistribution, supporting the notion that exosomes bearing CASK play an important role. The alterations to the actin cytoskeleton in the presence of CASK or exosomes bearing CASK are associated with a motile phenotype of podocytes.

This study was a prospective study designed to collect PBMC of patients. Because of the small number of patients enrolled in this study, the results should be validated on a larger cohort of patients. However, our results defined a secretory pathway of CASK and increased the level of evidence of immune cells implication in rFSGS. In conclusion, we report here the synthesis of CASK by the CD14^+^cells of rFSGS patients, and M2 polarized macrophages. These results highlighted the role of monocytes/macrophages in the pathology of rFSGS.

## Data Availability Statement

The datasets generated for this study are available on request to the corresponding author.

## Ethics Statement

The studies involving human participants were reviewed and approved by Comité Consultatif de Protection des Personnes participant à une Recherche Biomédicale ≫ (n°4/010). The patients/participants provided their written informed consent to participate in this study.

## Author Contributions

XZ performed the study and cell biology. AD designed the study and wrote the paper. SB was following patients. HL provided recombinant CASK and podocytes. AV performed western blotting. FH performed Flow cytometry.

## Conflict of Interest

The authors declare that the research was conducted in the absence of any commercial or financial relationships that could be construed as a potential conflict of interest.
